# Gender Differences in Predictors of Physical Activity among Korean College Students Based on the Health Promotion Model

**DOI:** 10.31372/20190401.1000

**Published:** 2019

**Authors:** Yeong-Ja Seo, Yeongmi Ha

**Affiliations:** a College of Nursing, Gyeongsang National University, Republic of Korea

**Keywords:** health promotion, physical activity, self-efficacy, gender

## Abstract

*Aim*: Despite the numerous physical and psychological benefits of physical activity (PA), the prevalence of achieving PA recommendations decreases in college students. Therefore, this study was conducted to identify factors influencing PA in male and female college students based on the Health Promotion Model.

*Methods*: A quantitative, cross-sectional research design was used to investigate factors influencing PA among male and female college students. A convenience sample of 264 Korean students was recruited from three colleges. Participants completed measures of physical activity, perceived benefits/barriers to PA, PA self-efficacy, activity-related affect, and peer support.

*Results*: A multiple regression analysis indicated that the factors affecting PA in male college students were PA self-efficacy and subjective economic status, while the factors affecting PA in female students were PA self-efficacy, subjective health status, activity-related affect, and peer support.

*Conclusions*: School health centers or wellness centers need to develop exercise self-efficacy enhancement programs to provide motivation for PA among college students. It might also be necessary to develop a customized PA program that considers gender differences.

## Introduction

The positive health effects of regular physical activity (PA) are well established, including the fact that engaging in regular PA could reduce the risk of mortality as well as chronic diseases such as stroke and cardiovascular disease, while also promoting mental health and quality of life (U.S. Department of Health and Human Services, 2018). Establishing a healthy lifestyle among college students is important because staying healthy will allow them to gain more knowledge and to participate in many activities. Despite the numerous benefits of PA, the prevalence of achieving PA recommendations decreases in college students undertaking tertiary education. A national Korean survey indicated that only 20.8% of college students engage in moderate-to-vigorous PA (Korea Centers for Disease Control and Prevention, 2016). Similarly, U.S. college students also have a low rate of participation in moderate-to-vigorous PA, at 21.2% (American College Health Association, 2014).

Gender differences exist in PA participation, which is generally higher among male as compared to female college students (Towne et al., 2017). Among the factors associated with PA, psychosocial factors such as self-efficacy, social support, and motivation have been used to explain the variation in PA between males and females. For example, motivation for exercise shows a gender difference. Male college students tend to exercise owing to intrinsic motivation including pleasure and challenge, whereas female college students tend to exercise because of extrinsic outcomes such as weight control (Choi, Chang, & Choi, 2015; Egli, Bland, Melton, & Czech, 2011). Therefore, when investigating predictors of PA, it is necessary to consider gender differences among college students related to the psychological and social factors affecting PA.

Health behavior theories provide an organized framework to understand key antecedents of behavior change. The Health Promotion Model (HPM) developed by Pender (1996) has frequently been used as a theoretical framework for measuring changes in PA and possible cognitive correlates. The HPM states that health behavior is primarily driven by behavior-specific cognitions and affect including the perceived benefits/barriers to action, perceived self-efficacy, activity-related affect, interpersonal influences, and situational influences (Pender, 1996). A growing body of research indicates that cognitive and affective factors, such as perceived benefits/barriers to action and perceived self-efficacy, are closely related to PA (Choi et al., 2015; Egli et al., 2011; Laird, Fawkner, Kelly, McNamee, & Niven, 2016; Mohamadian & Arani, 2014; Wu & Pender, 2005). In addition, several systematic reviews have shown that there are significant positive associations between peer support and PA, and that peer support could impact physical activity levels (Laird et al., 2016; Mendonça, Cheng, Mélo, & de Farias Júnior, 2014; Verloigne, Cardon, De Craemer, D’Haese, & De Bourdeaudhuij, 2016). For example, a systematic review of the influence of social support on physical activity found that social support provided by parents usually takes the form of buying sports equipment and providing transportation to sport venues, whereas friends tend to provide support by engaging in more vigorous activities and sports with each other (Mendonça et al., 2014).

Engaging in unhealthy behaviors places college students at risk for developing serious health problems later in life (Heller & Sarmiento, 2016; Hopper & Moninger, 2017; Tran et al., 2017). The transition from high school to college settings has been identified as a critical period for establishing health behaviors, as youth often start living away from their parents and experience more freedom and independence (Deforche, Van Dyck, Deliens, & De Bourdeaudhuij, 2015). College students have greater opportunities for establishing health-promoting behaviors and, on the other hand, may adopt irregular lifestyles with the abandonment of routines. Although identifying factors in promoting PA is critical, little is known about predictors of PA in relation to gender differences among college students. Furthermore, there is a lack of research on applications of the HPM to college students, although the HPM has been effectively applied with various other populations in previous studies. Therefore, this study aimed to identify factors influencing PA among male and female college students based on the HPM, by examining the relationships among individual characteristics, prior experiences of exercise, perceived benefits/barriers to PA, PA self-efficacy, activity-related affect, and peer support.

## Methods

### Study Design

A quantitative, cross-sectional research design was used to investigate factors influencing PA among male and female college students by examining relationships among individual characteristics, prior experiences of exercise, perceived benefits/barriers to PA, PA self-efficacy, activity-related affect, and peer support.

### Participants

The target population was full-time college students in urban settings, and a convenience sample of Korean students was recruited from three colleges. The inclusion criteria were as follows: students must be aged 18 to 30 years, enrolled in college, and willing to participate in the study. Students were excluded from participating if they were majoring in sports or physical education, owing to the large variation in PA that they would have compared with students in other majors.

The sample size was estimated using the G*power 3.1.9.2 program. The criteria for multiple regression were: statistical power (1 − *β*) of .80, significance level (*α*) = .05, medium effect size (*f*^2^) = .15, and number of predictors = 10. It indicated that we needed 118 participants. Allowing for attrition, we recruited a total of 270 students (135 male and 135 female). Questionnaires were distributed to these 270 college students, and all questionnaires were returned. Of these, six were excluded because of missing data, and therefore a total of 264 questionnaires were analyzed.

### Conceptual Framework

A conceptual framework was established based on the HPM and previous research. The third model of Pender’s HPM included three determinants of health-promoting behavior: individual characteristics and experiences, behavior-specific cognitions and affect, and behavioral outcomes. According to the HPM, there are unique personal characteristics and experiences that affect behavioral outcomes. Pender stated that behavior-specific cognitions and affect factors, such as perceived benefits/barriers to action and perceived self-efficacy, were closely related to health-promoting behavior.

In this study, subjective health status, socioeconomic status, and body perception were included to represent the individual characteristics because these factors were considered to be the psychological and sociocultural factors that affected a target behavior in the HPM. Since many studies have confirmed that the variables of behavior-specific cognitions and affect are significantly related to PA, these factors were also examined in this study. In addition, previous studies indicated that peer support might be an antecedent to increase PA self-efficacy. Finally, unique personal characteristics and experiences and behavior-specific cognitions and affect such as perceived benefits/barriers to action, perceived self-efficacy, activity-related affect, and peer support were included to identify factors affecting college students’ PA.

### Ethical Considerations

This study was approved by the Institutional Review Board of Gyeongsang National University, and then data were collected from three colleges. The purpose and procedures of the study were explained to the participants. All participants were informed that their participation was entirely voluntary, and they were free to withdraw from the research at any time without any loss of benefits to which they were entitled. Written consent was obtained from each participant if they agreed to take part. They were assured that their confidentiality and privacy would be maintained, and the collected data would be used only for research purposes.

### Instruments

**Individual characteristics.** The variables related to individual characteristics were composed of age, subjective economic status, subjective body perception, subjective health status, and prior exercise experiences answered by yes or no to see if they had regular PA during the last 6 months.

**Physical activity.** PA subscale of the Health-Promoting Lifestyle Profile II (HPLP-II) (Walker, Sechrist, & Pender, 1995) was used to measure the extent to which persons engage in a PA behavior. The instrument is an 8-item questionnaire with a 4-point Likert scale ranging from 1 (never) to 4 (routinely). Higher scores represent higher intensity of PA. The Cronbach’s alpha for this study was 0.80 for the PA subscale.

**Perceived benefits/barriers of PA.** Perceived benefits and barriers of PA were assessed using the Exercise Benefits/Barriers Scale (EBBS) (Sechrist, Walker, & Pender, 1987). The respondents are asked to rate their agreement or disagreement with statements about their perceptions about beneficial consequences and obstacles on a 4-point Likert scale ranging from 1 (strongly disagree) to 4 (strongly agree). Higher scores indicate greater perceived benefits and greater perceived barriers. The Cronbach’s alpha was 0.94 for perceived benefits of PA and 0.80 for perceived barriers of PA.

**Physical activity self-efficacy.** PA self-efficacy refers to self-confidence in the ability to adhere to PA in various situations. The 8-items of PA self-efficacy instrument developed by Kang and Gu (2006) is measured with a 5-point Likert scale ranging from 1 (very uncertain) to 5 (very certain). Higher values represent higher levels of self-efficacy for PA. The Cronbach’s alpha for this study was 0.86.

**Activity-related affect.** Activity-related affect is subjective feelings that occur during PA. This instrument is a 24-item consisting of positive (e.g., happy and exciting) adjectives, which are the scores measured by Korean Exercise Emotional Scale developed by Yoo and Kim (2002). Participants answered each item on a 5-point Likert scale ranging from 1 (do not feel) to 5 (very strongly feel). Higher scores indicate higher level of positive emotion associated with PA. The Cronbach’s alpha for this study was 0.96.

**Peer support.** Social support was defined as instrumental and emotional encouragement offered by others that acts as a sustaining resource for PA (Pender, 1996). The social support scale asked participants to answer how much support they receive from parents, siblings, and peers to increase their PA (Garcia et al., 1995). The social support subscale, adopted from the Child/Adolescent Exercise Social Support Scale (Garcia et al., 1995), was used to measure peer support regarding the frequency with which friends encouraged or praised them for engaging in PA. The scale is composed of 5-item, and a 3-point Likert scale ranging from 1 (never) to 3 (often), which higher scores mean higher peer support. The Cronbach’s alpha for this study was 0.74.

### Data Collection

The researcher approached college students at the entrance gate of the college, study lounge, and cafeteria near lecture halls. If interested in taking part, they were asked to respond to a verbal screening to determine their eligibility for this research. Two hundred and eighty-five students completed the verbal screening process, and 270 of them met eligibility criteria. Participation in the study was voluntary and students agreeing to participate were asked to sign a written informed consent form. A self-administered questionnaire written in Korean was distributed to each college student in September 2014. It took about 20 minutes to complete the questionnaire. A gift certificate of $5 was provided to each participant for participating in the survey.

### Data Analysis

The data were analyzed using the SAS 9.3 program (SAS Institute, Cary, NC). First, descriptive data (means and SDs) were used to examine scale distributions. Box plots, scatter plots, frequency, skewness, and kurtosis were used to examine normality assumptions and outliers for variables. The differences in PA according to individual characteristics and prior experiences (subjective economic status, subjective body perception, subjective health status, prior exercise experiences) in male and female college students were analyzed using *t*-tests or an analysis of variance (ANOVA). For a significant *F*-test, Scheffe’s post-hoc test was performed. Second, the correlations between PA, perceived benefits/barriers to PA, PA self-efficacy, activity-related affect, and peer support of male and female college students were analyzed with Pearson’s correlation coefficient. Prior to Pearson’s correlation analysis, a residual analysis was conducted to check the homoscedasticity assumption. The assumption for Pearson’s correlation was satisfied because the distribution of the residuals was approximately normal. Third, multiple regression was performed with PA of male and female college students as the dependent variable and individual characteristics, prior experiences of exercise, perceived benefits/barriers to PA, PA self-efficacy, activity-related affect, and peer support as independent variables. Nominal variables were dummy-coded to be included in the multiple regression analysis. Before the multiple regression analysis, multicollinearity was checked using variance inflation factors (VIF) less than 10, tolerances greater than .10, and a condition index less than 30. There were no multicollinearity diagnostics in this study: VIF (range 1.05–2.53), tolerance (range 0.39–0.94), and condition index (range 1.00–26.5).

## Results

### Individual Characteristics and Experiences of Male and Female College Students

The characteristics and experiences of male and female college students were represented in Table 1. A total of 264 students participated with 50.7% for male and 49.3% for female. The mean age of college students was 22.81 ± 2.35 for male and 21.46 ± 1.58 for female. In the subjective economic status, 60.4% of male and 72.3% of female students answered ‘middle’. Male college students were more likely to perceive as they had lean body, while female students were more likely to perceive as they were obese, and showed difference by gender. Most of male and female students reported ‘healthy or moderate’. Gender differences were found that 89.6% of male students and 50.8% of female college students answered that they had prior exercise experiences.

**Table 1:** Individual Characteristics and Experiences of Male and Female College Students (N = 264)

Variable	Male (*n* = 134)	Female (*n* = 130)
	*n*(%) or *M*(SD)	*n*(%) or *M*(SD)
Age (range 19–28 years)	22.81(2.35)	21.46(1.58)
Subjective economic status		
High	30(22.4)	9(6.9)
Middle	81(60.4)	94(72.3)
Low	23(17.2)	27(20.8)
Subjective body perception		
Lean	47(35.1)	18(13.8)
Appropriate	45(33.6)	55(42.3)
Obese	42(31.3)	57(43.9)
Subjective health status		
Healthy	100(74.6)	67(51.5)
Moderate	28(20.1)	52(40.0)
Unhealthy	6(4.5)	11(8.5)
Prior exercise experiences		
Yes	120(89.6)	66(50.8)
No	14(10.4)	64(49.2)

### Differences in PA according to Individual Characteristics and Experiences of Male and Female College Students

There were significant differences in PA according to subjective economic status (*F* = 3.40, *p* = .036), subjective body perception (*F* = 4.33, *p* = .015), subjective health status (*F* = 6.83, *p* = .001), and prior exercise experiences (*t* = 3.62, *p* = .001) in male students (Table 2). For female students, subjective health status (*F* = 8.42, *p* = .000) and prior exercise experiences (*t* = 2.69, *p* = .008) were statistically significant with PA.

**Table 2:** Differences of Physical Activity by Individual Characteristics and Experiences of Male and Female College Students (N = 264)

Variable	Male		Female	
	*M*(SD)	*t*(*p*) or *F*(*p*)	*M*(SD)	*t*(*p*) or *F*(*p*)
Subjective economic status		3.40(.036)^∗^		1.97(.143)
High^a^	2.41(0.61)	a > c	2.05(0.31)	
Middle^b^	2.23(0.55)		1.77(0.40)	
Low^c^	2.01(0.52)		1.81(0.45)	
Subjective body perception		4.33(.015)^∗^		2.72(.069)
Lean^a^	2.15(0.42)	b > c	1.59(0.40)	
Appropriate^b^	2.43(0.71)		1.84(0.37)	
Obese^c^	2.11(0.49)		1.82(0.43)	
Subjective health status		6.83(.001)^∗∗^		8.42(.000)^∗∗∗^
Healthy^a^	2.33(0.57)	a > b, c	1.92(0.41)	a > b, c
Moderate^b^	2.01(0.47)		1.70(0.35)	
Unhealthy^c^	1.68(0.25)		1.51(0.32)	
Prior exercise experiences		3.62(.001)^∗∗^		2.69(.008)^∗∗^
Yes	2.27(0.57)		1.89(0.40)	
No	1.87(0.36)		1.70(0.39)	

^∗^ *p* < .05.

^∗∗^ *p* < .01.

^∗∗∗^ *p* < .001.

### Correlations between Perceived Benefits/Barriers of PA, PA Self-efficacy, Activity-related Affect, Peer Support, and PA

Analysis of the Pearson’s correlations showed that PA of male college students had significant positive correlations with perceived benefits of PA (*r* = .318, *p* = .000), PA self-efficacy (*r* = .500, *p* < .0001), activity-related affect (*r* = .328, *p* = .000), and peer support (*r* = .379, *p* < .0001). And, there was a statistically significant negative correlation between perceived barriers and PA in male students (*r* = −.207,* p* = .016). In female college students, perceived benefits of PA (*r* = .355, *p* < .0001), PA self-efficacy (*r* = .550, *p* < .0001), activity-related affect (*r* = .476, *p* < .0001), and peer support (*r* = .364, *p* < .0001) were positively associated with PA. A negative relation was found between perceived barriers and PA (*r* = −.205,* p* = .018) (Table 3).

**Table 3:** Correlations between Perceived Benefits/Barriers of PA, PA Self-Efficacy, Activity-related Affect, Peer Support, and Physical Activity (N = 264)

	Variable	Perceived benefits *r*(*p*)	Perceived barriers *r*(*p*)	PA self-efficacy *r*(*p*)	Activity-related affect *r*(*p*)	Peer support *r*(*p*)	Physical activity *r*(*p*)
Male	Perceived benefits	1					
	Perceived barriers	.286^∗∗∗^	1				
	PA self-efficacy	.577^∗∗∗^	.381^∗∗∗^	1			
	Activity-related affect	.710^∗∗∗^	.257^∗∗^	.540^∗∗∗^	1		
	Peer support	.532^∗∗∗^	.400^∗∗∗^	.544^∗∗∗^	.528^∗∗∗^	1	
	Physical activity	.318^∗∗∗^	.207^∗^	.500^∗∗∗^	.328^∗∗∗^	.379^∗∗∗^	1
							
Female	Perceived benefits	1					
	Perceived barriers	.186^∗^	1				
	PA self-efficacy	.393^∗∗∗^	.342^∗∗∗^	1			
	Activity-related affect	.584^∗∗∗^	.118	.430^∗∗∗^	1		
	Peer support	.325^∗∗∗^	.229^∗∗^	.253^∗∗^	.316^∗∗∗^	1	
	Physical activity	.355^∗∗∗^	.205^∗^	.550^∗∗∗^	.476^∗∗∗^	.364^∗∗∗^	1

*Note*. PA = physical activity.

^∗^ *p* < .05.

^∗∗^ *p* < .01.

^∗∗∗^ *p* < .001.

### Predictors of PA in Male and Female College Students

As results of multiple regression analysis, it was found that PA self-efficacy (*β* = .391, *p* < .001), subjective economic status (*β* = .155, *p* < .05), explained male college students’ PA with 26.2% (*F* = 6.91, *p* < .0001). It was found that PA self-efficacy (*β* = .381, *p* < .001), subjective health status (*β* = .171, *p* < .05), activity-related affect (*β* = .166, *p* < .05), peer support (*β* = .166, *p* < .05), explained female college students’ PA with 39.5% (*F* = 11.5, *p* < .0001) (Table 4).

**Table 4:** Predictors of Physical Activity in Male and Female College Students (N = 264)

Variable	Male		Female	
	β	*t*	β	*t*
Individual characteristics and experiences				
Subjective economic status (reference = low)	.155	2.01^∗^	.035	0.49
Subjective health status (reference = unhealthy)	.101	1.21	.171	2.26^∗^
Prior exercise experiences (reference = no)	.015	0.20	.064	0.91
Behavior-specific cognitions and affect				
Perceived benefits of PA	.032	0.28	.033	0.38
Perceived barriers of PA	.041	0.50	.015	0.21
PA self-efficacy	.391	3.78^∗∗∗^	.381	4.63^∗∗∗^
Activity-related affect	.048	0.44	.223	2.50^∗^
Peer support	.117	1.19	.166	2.20^∗^
*F*(*p*)	6.91^∗∗∗^		11.5^∗∗∗^	
Adjusted *R*^2^	.262		.395	

Note. PA = physical activity.

^∗^ *p* < .05.

^∗∗^ *p* < .01.

^∗∗∗^ *p* < .001.

## Discussion

Health risk behaviors such as sedentary behavior and poor nutrition can be established during the transition from high school to college settings (Cullen et al., 1999; Deforche et al., 2015). Students need to be healthy and physically active in order to succeed in their academic performance. Practicing health-promoting behaviors, such as regular PA, is imperative for the college student population because engaging in PA can affect an individual’s lifelong health (Heller & Sarmiento, 2016; Hopper & Moninger, 2017). The present study provided a unique view of male and female college students’ PA by examining relationships between perceived benefits/barriers to PA, PA self-efficacy, activity-related affect, peer support, and PA based on the HPM. Identifying factors associated with PA in relation to gender differences at this critical time in a person’s life, could allow for targeted interventions, especially in college and university wellness centers, to improve the health and quality of life of college students.

PA self-efficacy was identified as having the strongest influence on PA of male and female college students in this study. A systematic review to identify factors influencing PA found that self-efficacy was shown to be the strongest factor impacting participation in PA (Choi, Lee, Lee, Kang, & Choi, 2017; Wu & Pender, 2005). Several previous studies demonstrated that PA self-efficacy is closely related to PA, thus supporting the results of the present study (Choi et al., 2015; Choi et al., 2017; Mohamadian & Arani, 2014; Wu & Pender, 2005). According to Bandura (1997), self-efficacy refers to optimistic self-beliefs that one can successfully perform the behavior required to obtain certain outcomes, and functions both directly and indirectly with health behavior change processes. In turn, self-efficacy is not only important for exercise initiation, but also for exercise maintenance and habituation (Bandura, 1997). As PA self-efficacy may provide motivation for PA, leading to actual activities, individuals with a high level of PA self-efficacy have confidence in their own ability to continue PA under any circumstances; thus, promoting PA self-efficacy in college students is very important. On the basis of this finding, college wellness centers and public health professionals need to develop exercise self-efficacy enhancement programs to provide motivation for PA among college students.

Having positive attitudes and beliefs about PA leads to a greater likelihood of undertaking and persisting with PA. Research shows that positive affect related to PA predicts exercise behaviors and intentions better than cognitive variables do (Helfer, Dlhai, & Geers, 2015; Kwan, Stevens, & Bryan, 2017; Rhodes & Kates, 2015). In the present study, activity-related affect was a significant contributing factor to PA for female but not male college students. This finding is consistent with the results of previous studies investigating the relationship between PA and affect in adults, especially females (Mohamadian & Arani, 2014; Williams, 2008). Recently, research has begun focusing on the role of affect in predicting PA initiation and continuation (Helfer et al., 2015; Kwan et al., 2017; Rhodes & Kates, 2015; Williams, 2008). Affective responses to a behavior may influence decisions regarding whether or not to repeat the behavior (Williams, 2008). Pleasure regarding PA participation can play an important role in accounting for exercise behavior. In this regard, affect-focused interventions for female college students could be designed to increase PA by enhancing positive feelings.

Peer support significantly influenced the PA of female college students in this study. Our results suggest that if females have active peers, do activity together with peers, and receive encouragement from their peers to be active, they may perceive more benefits related to PA, resulting in higher PA levels. A systematic review focusing on social support for PA demonstrated that social support from family and friends has been identified as a correlate of PA that can be used to inform interventions to enhance the PA level of females (Laird et al., 2016). Specifically, females’ peers can have an important effect on their PA because peers have the ability to foster females’ positive cognitions about, and enjoyment of, PA (Mohamadian & Arani, 2014; Verloigne et al., 2016). Engaging in PA with friends or peers can enhance cohesion and fellowship, which plays a crucial role in the continuation of successful PA in females (Verloigne et al., 2016). Thus, including the peer aspect in PA programs for female college students is effective in helping females to encourage one another to participate in PA.

Interestingly, male college students in this study showed increased PA when they perceived themselves as having high socioeconomic status. A literature review regarding socioeconomic determinants of PA, reported that convincing evidence supports a positive association between PA and socioeconomic status in adults (O’Donoghue et al., 2018). A possible explanation for the relationship between PA and socioeconomic status in male students is that people with higher socioeconomic status are more likely than those with lower socioeconomic status to participate in sports and activities (Eime, Charity, Harvey, & Payne, 2015; Puolakka et al., 2018).

Gender-based analyses reported that college men are more highly motivated in sport by intrinsic motives such as enjoyment and challenge, whereas women are more extrinsically motivated (Egli et al., 2011). Socioeconomic status influences PA in several ways. Generally, persons having a lower socioeconomic status are more likely to have restricted opportunities to participate in sports due to poor accessibility to sports facilities and the high burden of cost to buy sport equipment (Eime et al., 2015). Moreover, physical environment has an important influence on opportunities to be physically active, and usually persons with lower socioeconomic status have poorer access to physical environments where PA can take place such as parks, gardens, and stadiums (Chen et al., 2015; Choi et al., 2017). Therefore, for males of low socioeconomic status, the high costs of participating in PA and the lack of accessibility of sports facilities are important factors to address, as potential barriers to physical activity.

The strength of the present study is its use of the HPM to identify predictors of PA engagement among college students in relation to gender. This allows us to provide the most accurate information regarding influences on male and female college students’ PA. Despite this strength, there are some limitations that need to be acknowledged. Given the cross-sectional nature of this study, we were unable to draw causal relationships between PA and associated variables. Therefore, longitudinal or experimental studies would be needed to identify causal associations. Another limitation is the use of self-reporting survey questionnaires to estimate college students’ PA. Because subjective measurements of PA may decrease the reliability of study results, assessments using objective measurement indices are needed in the future along with a clear definition of PA. A final limitation is that our findings may not be generalizable to other college students due to our use of a convenience sample. Further, selection bias cannot be ruled out, due to the fact that students with health problems may have been less likely to be present at college when the data was collected.

## Conclusions

This study provides timely insights into behavior-specific cognitions and affect variables influencing PA among male and female college students. Our findings confirmed that factors affecting PA in males were PA self-efficacy and subjective economic status, while predictors of female students’ PA were PA self-efficacy, subjective health status, activity-related affect, and peer support.

Findings from this study suggest that future studies are needed to develop a school-based wellness program focusing on PA self-efficacy, given that this has the largest influence on college students’ PA, and because colleges and universities are an appropriate setting to promote sports activities. In addition, a customized exercise program considering gender differences should be implemented to help male and female college students engage in regular PA.

## Declaration of Conflicting Interests

The authors declared no potential conflicts of interest concerning the research, authorship, or publication of this article.
